# Population pharmacokinetics of caspofungin in critically ill patients receiving extracorporeal membrane oxygenation—an ASAP ECMO study

**DOI:** 10.1128/aac.01435-24

**Published:** 2024-12-18

**Authors:** Mohd H. Abdul-Aziz, Arne Diehl, Xin Liu, Vesa Cheng, Amanda Corley, Eileen Gilder, Bianca Levkovich, Shay McGuinness, Jenny Ordonez, Rachael Parke, Vincent Pellegrino, Steven C. Wallis, John F. Fraser, Kiran Shekar, Jason A. Roberts

**Affiliations:** 1 University of Queensland Centre for Clinical Research (UQCCR), Faculty of Medicine, The University of Queensland 1974 , Brisbane, Queensland, Australia; 2 Department of Clinical Pharmacy, Faculty of Pharmacy, Universiti Teknologi MARA 54703 , Puncak Alam, Malaysia; 3 Department of Intensive Care and Hyperbaric Medicine, The Alfred Hospital 5390 , Melbourne, Victoria, Australia; 4 School of Public Health and Preventive Medicine, Monash University 161667 , Melbourne, Victoria, Australia; 5 Critical Care Research Group and Adult Intensive Care Services, The Prince Charles Hospital 67567 , Brisbane, Queensland, Australia; 6 Cardiothoracic and Vascular Intensive Care Unit, Auckland City Hospital 58991 , Auckland, New Zealand; 7 Experiential Development and Graduate Education and Centre for Medicines Use and Safety, Faculty of Pharmacy and Pharmaceutical Sciences, Monash University 2541 , Melbourne, Victoria, Australia; 8 School of Nursing, The University of Auckland 56383 , Auckland, New Zealand; 9 Faculty of Medicine, The University of Queensland 1974 , Brisbane, Queensland, Australia; 10 Faculty of Health, Queensland University of Technology 1969 , Brisbane, Queensland, Australia; 11 Faculty of Health Sciences and Medicine, Bond University 104559 , Gold Coast, Queensland, Australia; 12 Herston Infectious Diseases Institute (HeIDI), Metro North Health, Brisbane, Queensland, Australia; 13 Department of Intensive Care Medicine and Pharmacy, Royal Brisbane and Women’s Hospital, Brisbane, Queensland, Australia; 14 Division of Anaesthesiology Critical Care Emergency and Pain Medicine, Nîmes University Hospital, University of Montpellier 27037 , Montpellier, France; University Children's Hospital Münster, Münster, Germany

**Keywords:** caspofungin, extracorporeal membrane oxygenation, population pharmacokinetics, critically ill patients

## Abstract

This study aimed to describe the population pharmacokinetics of caspofungin in critically ill patients receiving extracorporeal membrane oxygenation (ECMO) and to identify dosing regimens with a high likelihood of achieving effective exposures. Serial blood samples were collected over a single-dosing interval during ECMO. Total plasma concentrations were measured by a validated chromatographic assay. A population pharmacokinetic model was built and Monte Carlo dosing simulations were performed using Monolix. The probability of target attainment (PTA) and fractional target attainment (FTA) rates were simulated for various caspofungin dosing regimens against *Candida albicans*, *Candida glabrata*, and *Candida parapsilosis*. In all, 64 plasma concentration-time points were obtained from 8 critically ill patients receiving ECMO. Plasma concentration-time data for caspofungin were best described by a one-compartment model with first-order elimination. Lean body weight was identified as a significant covariate of volume of distribution. The typical volume of distribution and clearance of caspofungin in this cohort were 8.13 L and 0.55 L/h, respectively. The licensed caspofungin dosing regimen (a loading dose of 70 mg on day 1 followed by a maintenance dose of either 50 mg/day or 70 mg/day) demonstrated optimal PTA rates (≥90%) against *C. albicans* with an MIC of ≤0.064 mg/L, *C. glabrata* with an MIC of ≤0.125 mg/L, and *C. parapsilosis* with an MIC of ≤0.064 mg/L. The FTA analysis suggested that the licensed dosing regimen is only optimal (≥95%) against *Candida glabrata*, regardless of lean body weight. A higher-than-standard empirical dosing regimen (e.g., a loading dose of 100 mg on day 1, followed by a maintenance dose of 100 mg daily) is likely advantageous for critically ill patients receiving ECMO.

## INTRODUCTION

Extracorporeal membrane oxygenation (ECMO) is an established therapy in the management of acute cardiorespiratory failure in patients for whom maximal medical management fails. The primary goal of ECMO is to ensure adequate oxygen delivery to tissues while the underlying cause of cardiorespiratory failure is being assessed and treated. The use of ECMO in the adult intensive care unit (ICU) has increased in the last 10 years (1, 2), particularly during the recent coronavirus disease (COVID-19) pandemic. Despite its life-saving potential, the use of ECMO is not without inherent risks and complications. Critically ill patients who receive ECMO are at a greater risk of developing nosocomial infections, including those caused by fungal pathogens (*Candida* spp.), which are associated with increased morbidity and mortality (3). Therefore, effective antifungal therapy in this patient population is essential.

Caspofungin is an important antifungal option for critically ill patients with suspected or proven invasive candidiasis (4). In the context of providing effective caspofungin therapy for these patients, the use of ECMO introduces a new challenge for clinicians as this device is hypothesized to alter drug concentrations and consequently, dosing requirements in critically ill patients. *Ex vivo* and *in vivo* animal data have shown that highly protein-bound drugs, such as caspofungin, are more prone to circuit drug loss and sequestration when compared with others (5–7). However, there are few clinical pharmacokinetic data and no population pharmacokinetic data available to guide effective caspofungin dosing in critically ill patients while receiving ECMO.

This paper aimed to describe the population pharmacokinetics of caspofungin and sources of pharmacokinetic variability in critically ill patients receiving ECMO. With the resulting population pharmacokinetic model, we then sought to perform Monte Carlo dosing simulations to identify caspofungin dosing regimens likely to achieve exposures associated with maximal efficacy.

## MATERIALS AND METHODS

### Setting

The present analysis is part of the ASAP ECMO study program that was conducted from November 2012 to November 2019 (8). The ASAP ECMO study was a prospective, open-labeled multicenter pharmacokinetic study performed in six ICUs across Australia, New Zealand, South Korea, and Switzerland. A detailed study protocol has been previously described elsewhere (8, 9), and as such, is only briefly outlined in this paper.

### Study population

Patients were eligible for recruitment if they were adult (18–90 years old) ICU patients who were receiving caspofungin while undergoing ECMO therapy for cardiac and/or respiratory failure. Patients were excluded if they required massive blood transfusion (i.e., >50% of blood volume in the preceding 8 hours), received therapeutic plasma exchange in the preceding 24 hours, had bilirubin >150 µmol/L, were pregnant, or were known to be allergic to caspofungin.

### Caspofungin administration and ancillary treatments

Caspofungin was reconstituted and administered as an intermittent intravenous infusion in accordance with the standard administration protocol at each participating center. Caspofungin dosing regimens, ECMO modalities/configurations, and all other subsequent patient management (e.g., RRT application) were determined by the treating clinician in accordance with local clinical guidelines and practices.

### Study procedures

Pharmacokinetic sampling was performed during a single dosing interval once the patient had been stabilized on ECMO. Blood samples were drawn from an existing arterial line and were collected into lithium-heparinized tubes. Blood samples were collected at 0, 0.25, 0.5, 0.75, 1, 1.5, 2, 3, 6, 8, 12, and 24 hours after commencement of infusion.

The blood samples were centrifuged at 3,000 × *g* for 10 minutes to obtain plasma, which was then frozen at –80°C and stored locally at participating sites. The frozen plasma samples were shipped on dry ice by a commercial courier company to the central bioanalysis laboratory at the University of Queensland Centre for Clinical Research (UQCCR) for bioanalysis.

Relevant demographic, anthropometric, clinical, and treatment-related data were collected on the day of pharmacokinetic sampling. The dosing regimen of caspofungin (the dose, duration of infusion, and frequency of administration) and the exact times of dosing and pharmacokinetic sampling were recorded. Each participating site maintained an electronic database for their participants that was later merged into a central database.

### Caspofungin assay

Total caspofungin concentrations in plasma were measured by a validated ultra-high performance liquid chromatography method on a Nexera X2 system connected to a Shimadzu 8030 + triple quadrupole mass spectrometer (Shimadzu Corporation, Kyoto, Japan). Dicloxacillin was used as the internal standard. The assay was performed in batches, and samples were analyzed concurrently with calibration standards and quality-control replicates at high (18 mg/L), medium (3 mg/L), and low (0.6 mg/L) concentrations. The assay limit for caspofungin in plasma was 0.1 mg/L and linearity was established within a range of 0.1–20 mg/L. Precision and accuracy at three different concentrations were all within 12%. Bioanalysis methods were performed in accordance with the U.S. Food and Drug Administration’s guidance for industry on bioanalysis (10).

### Population pharmacokinetic analysis

#### Structural model development

Plasma concentration-time data for caspofungin were analyzed using a nonlinear mixed-effect modeling approach in Monolix version 2021R2 (Lixoft, Antony, France) implementing the stochastic approximation expectation maximization (SAEM) algorithm. Plasma concentration-time data for caspofungin were fitted to one- and two-compartment disposition models with first-order elimination. Between-subject variability (BSV or ω) was described using an exponential model:


$$\theta_{j}=\theta_{p}\times exp\left(\eta_{j}\right)$$

where *θ_j_* is the estimate for a pharmacokinetic parameter in the *j^th^* patient as predicted by the model, *θ_p_* is the typical population pharmacokinetic parameter value, and *η_j_* is a random variable from a normal distribution with zero mean and variance *ω*^2^, which is an estimated value. Additive, proportional, and combined residual random error models were tested to describe residual unexplained variability (ε). Individual estimates for pharmacokinetic parameters were assumed to follow a log-normal distribution.

Competing models were evaluated based on visual inspection of goodness-of-fit (GOF) plots, as well as numerical assessment of objective function value (OFV) and the corrected Bayesian information criterion (BICc). A reduction in the OFV of >3.84 for one degree of freedom was considered a statistical improvement (*P* < 0.05) for a model.

#### Covariate screening and model development

The effects of the following covariates on caspofungin pharmacokinetic parameters were evaluated: age, sex, body weight measures (including ideal body weight (11), adjusted body weight (12), and lean body weight [LBW] (13)), body mass index, estimated Cockcroft-Gault creatinine clearance (CL_cr_) (14), serum albumin and bilirubin, blood urea nitrogen, Acute Physiology and Chronic Health Evaluation (APACHE) II (15) and Sequential Organ Failure Assessment (SOFA) (16) scores, receipt of RRT, and ECMO treatment variables (i.e., duration, mode, and flow rate). Continuous covariates were centered on their median values and categorical covariates (e.g., sex, receipt of RRT, and ECMO mode) were tested using a linear model. Stepwise forward inclusion and backward elimination approaches were employed during a covariate model-building process. Reductions in OFV of >3.84 (*P* < 0.05) and >6.63 (*P* < 0.01) were required for a covariate to be considered in the forward inclusion and backward elimination steps, respectively.

#### Model evaluation and prediction

Observed versus individual-predicted and population-predicted plots, individual-weighted residual versus individual-predicted and time plots (IWRES), and normalized prediction distribution error (NPDE) versus population-predicted and time plots were used to evaluate the graphical GOF of competing models. A visual predictive check (VPC) was performed by simulating 500 patients to assess the predictive performance of the final model. Visual checks were performed by comparing the observed data points with the 90% confidence intervals of the simulated 5th, 50th, and 95th percentile curves. The final model accuracy and stability were also examined using a bootstrap method—a 1,000-run bootstrap resampling procedure was performed in Monolix using the Rsmlx package (R Speaks “Monolix” version 4.0.2) in R software (version 4.1.3). The median, 2.5%, and 97.5% values obtained from the 1,000 bootstrap runs for each parameter estimate were compared with the parameter estimates of the final model.

#### Dosing simulations

Different caspofungin dosing regimens were evaluated using Monte Carlo simulation in Simulx (Lixoft, Antony, France). The parameter estimates of the final pharmacokinetic model were used to simulate 1,000 plasma concentration-time profiles for the following caspofungin dosing regimens (as 1 hour of intravenous infusion): (i) loading dose of 50, 70, or 100 mg followed by a maintenance dose of 50 mg; (ii) loading dose of 70, 100, or 150 mg followed by a maintenance dose of 70 mg; and (iii) loading dose of 100, 150, or 200 mg followed by a maintenance dose of 100 mg. Simulations were performed for a typical patient with an LBW of 40, 50, 60, or 80 kg. For each dosing regimen, the probability of target attainment (PTA) was calculated as the percentage of patients achieving the target ratio of total drug area under the concentration-time curve during 24 hours over a given minimum inhibitory concentration (AUC_0–24_/MIC) at 48 hours of therapy. PTA was calculated for MIC of 0.004, 0.016, 0.032, 0.064, 0.125, and 1 mg/L. As suggested by *in vivo* animal model experiments of Andes et al. (17), the target AUC_0–24_/MIC ratios were ≥865 for *Candida albicans*, ≥450 for *Candida glabrata*, and ≥1185 for *Candida parapsilosis*. A dosing regimen was considered “optimal” if the PTA was ≥90%. Caspofungin MIC breakpoints against *Candida* spp. have not yet been established. According to the European Committee on Antimicrobial Susceptibility Testing (EUCAST) recommendations, isolates susceptible to anidulafungin and micafungin should be considered susceptible to caspofungin as well (18). The EUCAST MIC breakpoint for anidulafungin is 0.03 mg/L for *Candida albicans*, 0.06 mg/L for *Candida glabrata*, and 4 mg/L for *Candida parapsilosis* (18).

Fractional target attainment (FTA) was calculated by comparing the PTA against reported MIC distributions for *C. albicans*, *C. glabrata*, and *C. parapsilosis* (19, 20). A dosing regimen was considered “optimal” if the FTA was ≥95%.

## RESULTS

### Study population characteristics

A total of 64 plasma concentration-time points were obtained from eight critically ill patients while receiving ECMO support. The median time to pharmacokinetic sampling after ECMO initiation was 3.5 days (range: 1–30). The baseline demographic and clinical characteristics of the study population are presented in Table 1. Most patients (75.0%) received veno-venous ECMO support. Five patients (62.5%) received concomitant renal replacement therapy during pharmacokinetic sampling, with the majority supported by continuous veno-venous hemodiafiltration. All patients received a loading dose of 70 mg (as 1 hour of infusion) on day 1 of caspofungin therapy, which was then followed by a maintenance dose of either 50 mg (6 patients) or 70 mg (2 patients) daily.

**TABLE 1:** Clinical and demographic characteristics of the recruited patients

Patient characteristic	All patients (*n* = 8)
Age (in years)	36.5 (20.0–62.0)
Actual bodyweight (in kg)	84.0 (55.0–130.0)
Ideal bodyweight (in kg)	68.0 (55.0–80.0)
Lean body weight (in kg)	58.9 (37.3–81)
Body mass index (in kg/m^2^)	25.8 (19.5–38)
Male, n (%)	5 (62.5)
APACHE II (on admission)	16.5 (10–32)
SOFA score (on sampling day)	8.0 (5.0–15.0)
Estimated creatinine clearance (in mL/min)	57.0 (31.0–260.0)
Blood urea nitrogen, mmol/L	9.8 (3.4–24.3)
Serum albumin, g/L	24.0 (17.0–33.0)
Total protein, g/L	58.5 (47.0–79.0)
Total bilirubin, µmol/L	12.0 (10.0–27.0)
ECMO indication, n (%)	
Acute respiratory distress syndrome	3 (37.5)
Cardiac arrest	1 (12.5)
Cardiogenic shock	1 (12.5)
Lung transplant	3 (37.5)
ECMO mode, n (%)	
Veno-arterial	2 (25.0)
Veno-venous	6 (75.0)
ECMO flow rate	3.5 (2.9–5.7)
Renal replacement therapy, n (%)	5 (62.5)
CVVHD	4 (80.0)
SLED	1 (20.0)

### Population pharmacokinetic model building

Plasma concentration-time data for caspofungin were best described by a one-compartment model with first-order elimination. A two-compartment model was not selected because it did not improve the model fit (no decrease in BICc). Residual unexplained variability was described by an additive error model.

Initial screening suggested that sex, lean body weight, and serum albumin could be potential covariates for pharmacokinetic parameters. The influence of sex and lean body weight was evaluated on the volume of distribution, and the influence of serum albumin was evaluated on clearance. Only the inclusion of lean body weight on the volume of distribution improved the model fit, and the relationship was best expressed as below:


$$V=Vpop\;\times \;{\left(\frac{LBW}{58.9}\right)}^{1.24}$$

where *V* is the estimated volume of distribution in a given individual (in L), *V*_pop_ is the typical value of caspofungin volume of distribution in the population, and LBW is the individual’s estimated lean body weight (in kg) based on Janmahasatian et al. equation (13).

**Fig 1:**
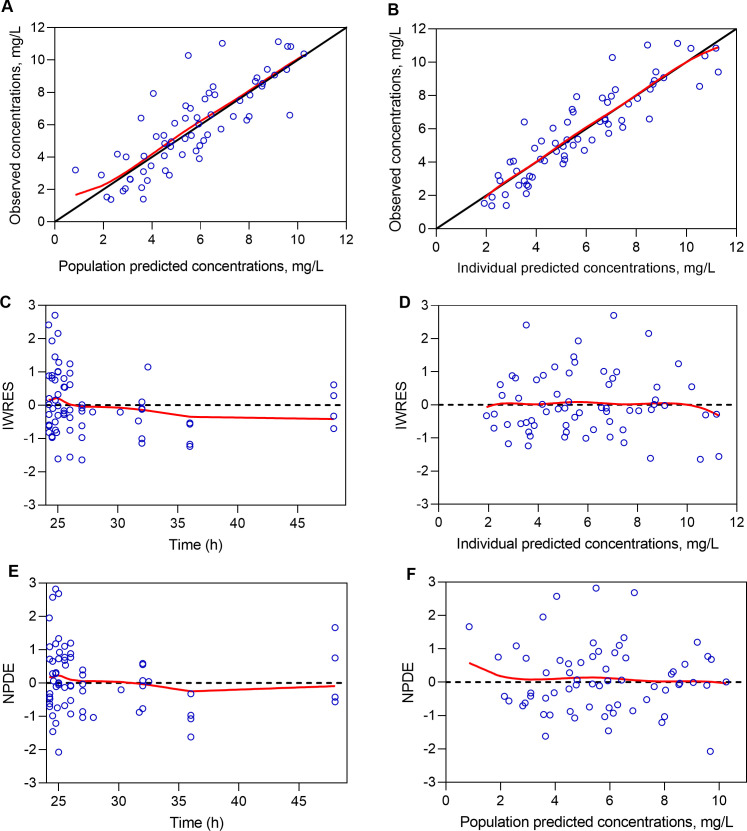
Goodness-of-fit plots associated with the final population pharmacokinetic model for caspofungin in critically ill patients receiving extracorporeal membrane oxygenation. (**A**) Observed versus population predicted concentrations; (**B**) observed versus individual predicted concentrations; (**C**) IWRES versus time; (**D**) IWRES versus predicted concentrations; (**E**) NPDE versus time; and (**F**) NPDE versus population predicted concentrations. The solid red line represents the spline line.

**Fig 2:**
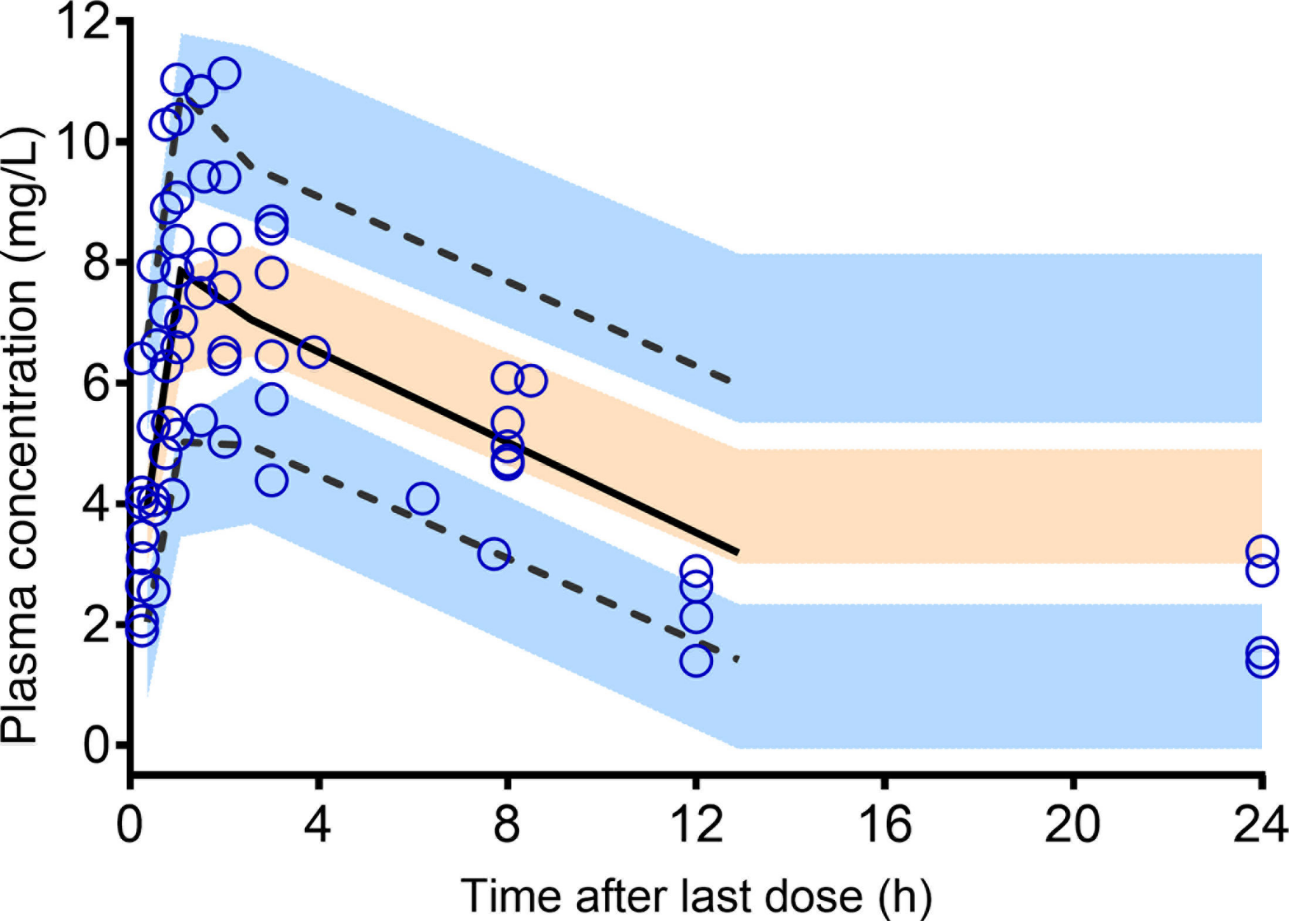
Visual predictive check plot associated with the final population pharmacokinetic model for caspofungin. The circles represent the observed data. The solid black line represents the 50th percentile of the simulated data, and the dashed black lines represent the 5th and 95th percentiles of the simulated data. The shaded areas represent the 95% confidence intervals of the 5th, 50th, and 95th percentiles of the simulated data.

The pharmacokinetic model-building process is further detailed in Supplementary materials. Typical pharmacokinetic parameter estimates from the final model are summarized in Table 2. The goodness-of-fit (Fig. 1) and visual predictive check (Fig. 2) plots demonstrated that the final model adequately described the concentration-time data for caspofungin. Furthermore, the bootstrap results showed median values that closely aligned with the typical pharmacokinetic parameter estimates of the final model (Table 2), with narrow 95% confidence intervals.

**TABLE 2:** Typical population pharmacokinetic parameter estimates of the final model and the 1,000 bootstrap runs^*a*^

Parameter	Estimate (%RSE)	Bootstrap median (95% CI)
Fixed effect		
*CL* (L/h)	0.55 (11.9)	0.55 (0.42–0.68)
*V* (L)	8.13 (7.15)	8.27 (7.07–10.8)
LBW effect on *V*	1.24 (23.3)	1.24 (0.08–1.91)
Between-subject variability		
*CL* (%)	25.5 (34.3)	21.3 (2.86–34.9)
*V* (%)	7.41 (101)	4.94 (1.03–22.6)
Residual error		
Additive (mg/L)	1.20 (10.0)	1.17 (0.85–1.47)

^*a*^ CI = confidence interval; CL = clearance; LBW = lean body weight; RSE = relative standard error; V = volume of distribution.

### Dosing simulations

**Fig 3:**
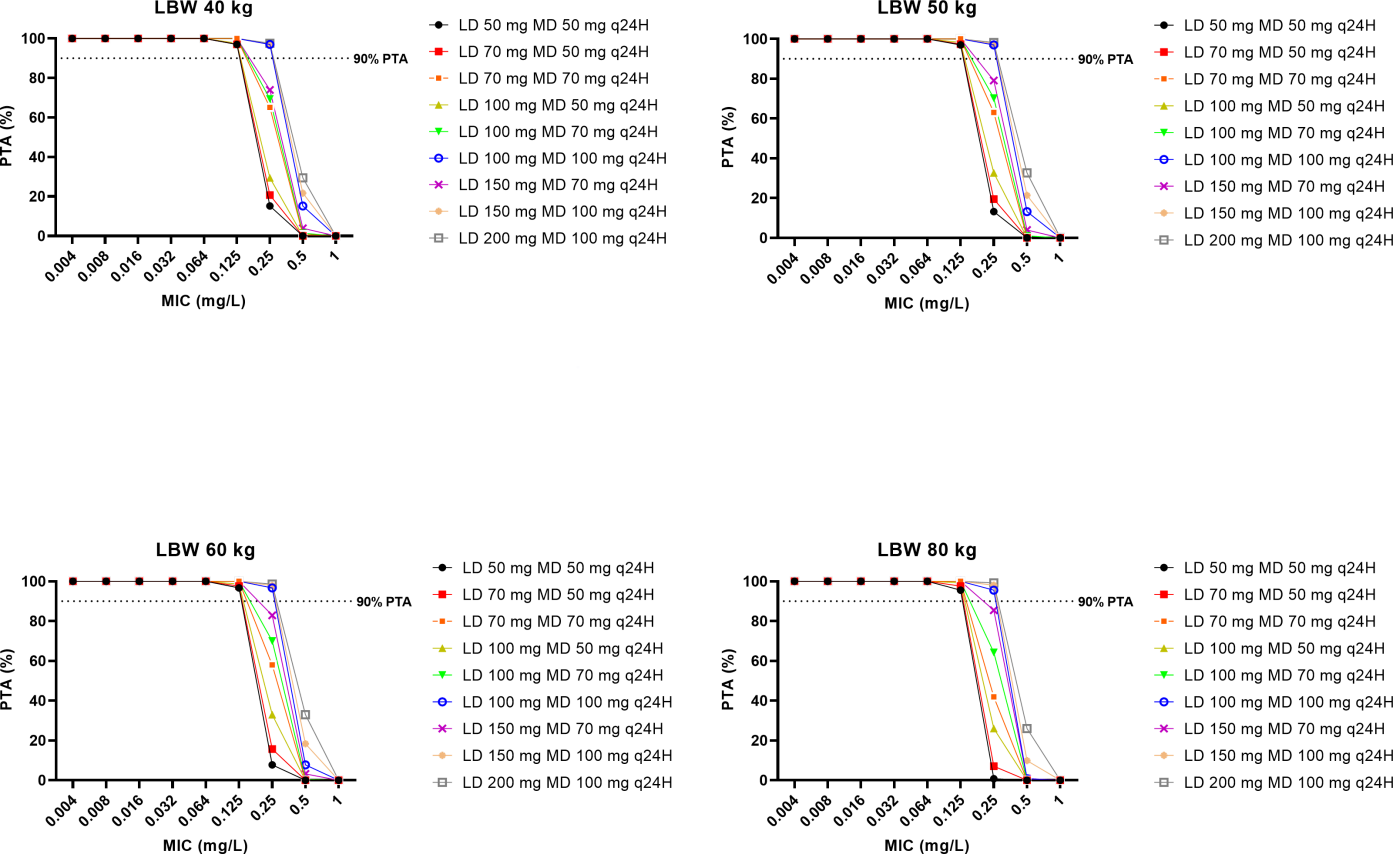
The PTA on Day 2 for simulated caspofungin dosing regimens to achieve AUC_0–24_/MIC ratio of ≥450 for *Candida glabrata* in patients with an LBW of 40 kg, 50 kg, 60 kg, and 80 kg. The dashed black lines denote optimal dosing regimen, that is, achieving ≥90% PTA.

**Fig 4:**
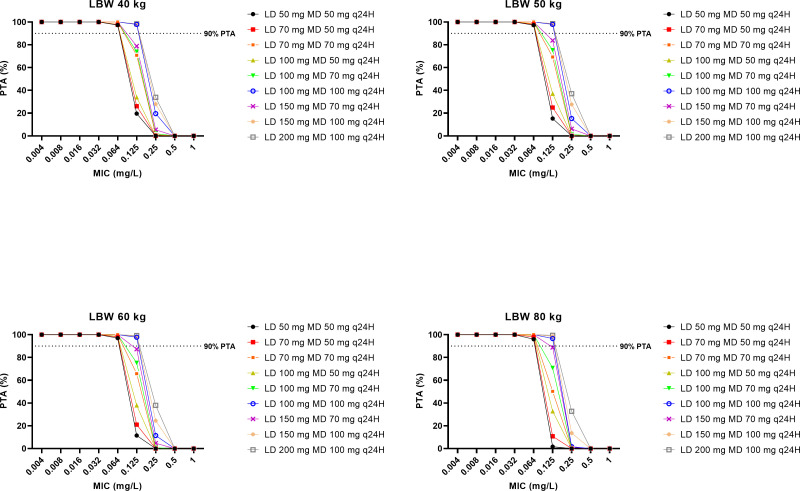
The PTA on Day 2 for simulated caspofungin dosing regimens to achieve AUC_0–24_/MIC ratio of ≥865 for *Candida albicans* in patients with a LBW of 40 kg, 50 kg, 60 kg, and 80 kg. The dashed black lines denote optimal dosing regimen, that is, achieving ≥90% PTA.

**Fig 5:**
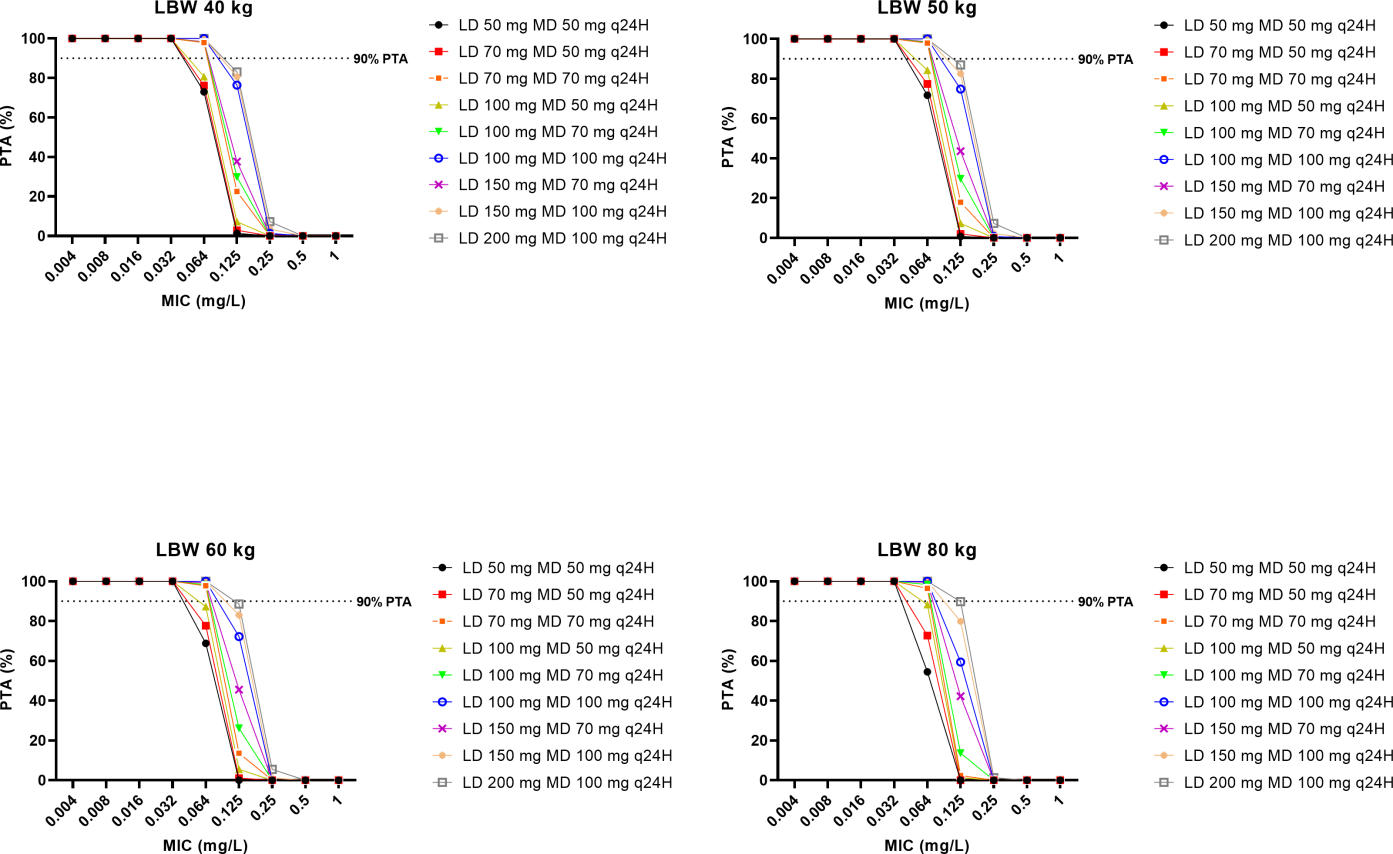
The PTA on Day 2 for simulated caspofungin dosing regimens to achieve AUC_0–24_/MIC ratio of ≥1,185 for *Candida parapsilosis* in patients with a LBW of 40 kg, 50 kg, 60 kg, and 80 kg. The dashed black lines denote the optimal dosing regimen, that is, achieving ≥90% PTA.

The PTAs for various caspofungin dosing regimens across different scenarios against *C. albicans*, *C. glabrata*, and *C. parapsilosis* are presented in Fig. 3 to 5, respectively. For *C. albicans* and *C. glabrata*, the licensed caspofungin dosing regimen (an initial loading dose of 70 mg on day 1 followed by a maintenance dose of either 50 mg/day or 70 mg/day) demonstrated optimal rates of PTA against strains with a MIC of ≤0.064 mg/L and ≤0.125 mg/L, respectively. For *C. parapsilosis*, a maintenance dose of 50 mg/day and 70 mg/day demonstrated optimal rates of PTA against strains with a MIC of ≤0.032 mg/L and ≤0.064 mg/L, respectively. The use of a higher loading dose in a dosing regimen improved the PTAs across all simulated scenarios.

The FTAs for various caspofungin dosing regimens against *Candida* spp. are summarized in Table 3. The licensed caspofungin dosing regimen was observed to be optimal against *C. glabrata* during the first 48 hours of therapy. An initial loading dose of 100 mg on day 1 followed by a maintenance dose of 100 mg daily was optimal against *C. albicans* during the first 48 hours of therapy for patients with a lean body weight of 40–60 kg. For patients with a lean body weight of 80 kg, an initial loading dose of at least 150 mg on day 1 followed by 100 mg daily was optimal against *C. albicans* during the first 48 hours of therapy. None of the simulated dosing regimens was optimal against *C. parapsilosis* during the first 48 hours of therapy.

**TABLE 3:** Fractional target attainment for different dosing regimens against *Candida glabrata*, *Candida albicans*, and *Candida parapsilosis^a^^,^^b^*

Dosing regimen	LBW (kg)	*Candida glabrata*		*Candida albicans*		*Candida parapsilosis*	
LD 50 mg MD 50 mg	40	Day 1	Day 2	Day 1	Day 2	Day 1	Day 2
	50	**+**	**+**	−	−	−	−
	60	**+**	**+**	−	−	−	−
	80	−	**+**	−	−	−	−
LD 70 mg MD 50 mg	40	**+**	**+**	−	−	−	−
	50	**+**	**+**	−	−	−	−
	60	**+**	**+**	−	−	−	−
	80	**+**	**+**	−	−	−	−
LD 100 mg MD 50 mg	40	**+**	**+**	**+**	−	−	−
	50	**+**	**+**	**+**	−	−	−
	60	**+**	**+**	**+**	−	−	−
	80	**+**	**+**	−	−	−	−
LD 70 mg MD 70 mg	40	**+**	**+**	−	−	−	−
	50	**+**	**+**	−	−	−	−
	60	**+**	**+**	−	−	−	−
	80	**+**	**+**	−	−	−	−
LD 100 mg MD 70 mg	40	**+**	**+**	**+**	−	−	−
	50	**+**	**+**	**+**	−	−	−
	60	**+**	**+**	**+**	−	−	−
	80	**+**	**+**	−	−	−	−
LD 150 mg MD 70 mg	40	**+**	**+**	**+**	−	−	−
	50	**+**	**+**	**+**	−	−	−
	60	**+**	**+**	**+**	−	−	−
	80	**+**	**+**	**+**	−	−	−
LD 100 mg MD 100 mg	40	**+**	**+**	**+**	**+**	−	−
	50	**+**	**+**	**+**	**+**	−	−
	60	**+**	**+**	**+**	**+**	−	−
	80	**+**	**+**	−	**+**	−	−
LD 150 mg MD 100 mg	40	**+**	**+**	**+**	**+**	−	−
	50	**+**	**+**	**+**	**+**	−	−
	60	**+**	**+**	**+**	**+**	−	−
	80	**+**	**+**	**+**	**+**	−	−
LD 200 mg MD 100 mg	40	**+**	**+**	**+**	**+**	**+**	−
	50	**+**	**+**	**+**	**+**	**+**	−
	60	**+**	**+**	**+**	**+**	−	−
	80	**+**	**+**	**+**	**+**	−	−

^*a*^ LBW = lean body weight; LD = loading dose; MD = maintenance dose.

^*b*^ + indicates ≥95% fractional target attainment against *Candida* spp.

## DISCUSSION

Earlier preclinical studies showed that caspofungin was prone to circuit sequestration, potentially due to its high protein-binding nature (6, 7). The present study evaluated ECMO and ECMO-related variables as potential covariates but did not find any significant effect on caspofungin pharmacokinetic parameters. Our findings further corroborate the notion that the use of ECMO had negligible impact on the pharmacokinetics of caspofungin in critically ill patients (21–24). In addition to this, other clinical studies have also observed the effect of ECMO on the pharmacokinetics of other echinocandins to be minimal (25, 26). Furthermore, the typical volume of distribution and clearance of caspofungin in this cohort are generally consistent with other previously published data on critically ill patients with or without ECMO support (21–24, 27–33). The typical volume of distribution and clearance of caspofungin described in other studies ranged between 6.19 to 20.91 L and 0.27 to 0.98 L/h, respectively. However, direct comparison between our parameter estimates and those from other studies is difficult as most studies used a two-compartment model to describe caspofungin pharmacokinetics. Consistent with previous studies, we found that the licensed caspofungin dosing regimen may not be optimal for critically ill patients with or without ECMO support, particularly early in the course of therapy. A higher-than-standard empirical dosing regimen, for example, an initial loading dose of 100 mg on day 1, followed by a maintenance dose of 100 mg daily, will improve attainment of target exposures for empirical caspofungin therapy when susceptible *Candida* spp. is suspected in critically ill patients receiving ECMO.

In the present study, lean body weight was identified as a significant covariate of caspofungin volume of distribution. This observation is in agreement with earlier pharmacokinetic studies (27–37), which have also highlighted the influence of body size descriptors on caspofungin pharmacokinetics and dosing requirements. The increase in total body weight (28, 29, 31, 32, 34, 35) and fat-free mass (27) were associated with higher volume of distribution and clearance of caspofungin in previous studies. Several studies have reported that this phenomenon also led to a decrease in both caspofungin concentrations (35, 37) and AUC exposures (34, 35). This has direct implications for dosing, particularly for overweight and obese patients since the AUC/MIC ratio drives caspofungin’s activity (17). The product information recommends an initial loading dose of 70 mg on day 1 and a maintenance dose of 50 mg/day thereafter. For patients whose body weight is greater than 80 kg, the maintenance dose should be increased to 70 mg/day. However, Martson et al. recently demonstrated that the licensed caspofungin dosing regimen is unlikely to be optimal for 25% of patients with a low body weight (<50 kg), for 80% of patients with an average body weight (~80 kg), and for all overweight patients (≥120 kg). In the present study, the median AUC_0 – 24_ exposure was 115 mg·h/L (range: 81–151). This AUC_0 – 24_ exposure was comparable to those reported in ICU patients (74–135 mg·h/L) (27, 29, 34, 38–40) but was higher than those established in healthy volunteers (98 mg·h/L) (41, 42). Current pharmacokinetic data suggest using either an alternative dosing approach (e.g., weight-based dosing) or a higher-than-standard caspofungin dosing regimen to achieve exposures associated with maximal caspofungin efficacy.

Previous studies have rarely evaluated the correlation between lean body weight and caspofungin pharmacokinetic parameters. The present study showed that lean body weight, based on Janmahasatian et al. equation. (13), accounted for approximately 25% of the variability in caspofungin volume of distribution. Although the Janmahasatian et al. equation (13). was developed to estimate fat-free mass, the estimate is considered to be a representation of lean body weight and is commonly used interchangeably. The equation also addresses a common limitation associated with other equations, where the estimation of lean body weight can be unreliable for patients at the extremes of size. Our study presents an alternative and interesting approach to guide caspofungin dosing for critically ill patients receiving ECMO.

It is challenging to ascertain the optimal dosing regimen for caspofungin based on MIC breakpoints. The European Committee on Antimicrobial Susceptibility Testing (EUCAST) MIC breakpoints have yet to be established for caspofungin against *Candida* spp. due to significant interlaboratory variation in MIC reporting (18). Until this problem is resolved, Candida isolates that are susceptible to anidulafungin and micafungin should also be considered susceptible to caspofungin. In our dosing simulations (Fig. 3 and 4), the licensed caspofungin dosing regimen achieved optimal PTA rates against *C. albicans* and *C. glabrata* when considering MICs of 0.03 mg/L and 0.06 mg/L, respectively (i.e., EUCAST MIC breakpoints for anidulafungin against *C. albicans* and *C. glabrata*). However, it is rare to know the MIC of the offending pathogen at the start of the treatment. Therefore, rapid and adequate caspofungin exposure is necessary to cover for most susceptible *Candida* spp. Our FTA simulations indicated that an initial loading dose of 100 mg on day 1, followed by a maintenance dose of 100 mg daily is likely advantageous for empirical caspofungin therapy for patients with a lean body weight of 40–60 kg when susceptible *Candida* spp. is suspected (Table 3). For patients with a lean body weight of 80 kg, an initial loading dose of at least 150 mg on day 1 followed by 100 mg daily is likely required. As none of the simulated dosing regimens were optimal against *C. parapsilosis*, an even higher dosing regimen is likely needed when this species is involved. To the best of our knowledge, this is the fifth study recommending higher-than-recommended loading doses of caspofungin to achieve optimal exposures rapidly in critically ill patients in the ICU (27, 28, 31, 32). Given that caspofungin has been demonstrated to be safe at doses of up to 200 mg/day (22, 43–45), such a dosing strategy can be considered for this patient population and should now be evaluated in a large clinical trial to assess its impact on patient outcomes. For centers with therapeutic drug monitoring (TDM) capabilities, TDM-guided dosing is likely the safest and most effective way to address this uncertainty, although current guidelines do not advocate for echinocandin TDM at this time (46, 47).

This study has several limitations. First, the sample size was small and may not have been sufficient to characterize the pharmacokinetic heterogeneity of caspofungin in critically ill patients receiving ECMO. However, this study addresses the current gap in the literature by being the first to provide caspofungin dosing recommendations for patients whilst on ECMO support. Second, since the relationship between caspofungin exposures and optimal clinical outcomes has not been established, we used the best available data linking exposures to maximal caspofungin activity from *in vivo* animal PK/PD models (17). Third, although ECMO appears to have a negligible impact on caspofungin pharmacokinetics in the present study, the true influence may not be able to be fully quantified without non-ECMO controls for comparison. Fourth, in contrast to previous studies (28, 29, 31, 37), we did not find a significant relationship between albumin concentrations and the pharmacokinetics of caspofungin. This could be due to small variations in albumin concentrations as all patients demonstrated some degree of hypoalbuminemia (range 17–33 g/L).

### Conclusions

The licensed caspofungin dosing regimen may not be optimal for critically ill patients receiving ECMO, particularly early in the course of therapy. An initial loading dose of 100 mg on day 1, followed by a maintenance dose of 100 mg daily is likely advantageous for empirical caspofungin therapy for patients with a lean body weight of 40–60 kg when susceptible *Candida* spp. is suspected.
